# Reinforcement learning using Deep $Q$ networks and $Q$ learning accurately localizes brain tumors on MRI with very small training sets

**DOI:** 10.1186/s12880-022-00919-x

**Published:** 2022-12-23

**Authors:** J. N. Stember, H. Shalu

**Affiliations:** 1 Department of Radiology, Memorial Sloan Kettering Cancer Center, 1275 York Avenue, Box 29, New York, NY 10065 USA; 2 Department of Aerospace Engineering, Indian Institute of Technology Madras, Chennai, 600 036 India

**Keywords:** Deep reinforcement learning, Reinforcement learning, Gridworld, Localization, Regression, Brain tumors

## Abstract

**Background:**

Supervised deep learning in radiology suffers from notorious inherent limitations: 1) It requires large, hand-annotated data sets; (2) It is non-generalizable; and (3) It lacks explainability and intuition. It has recently been proposed that reinforcement learning addresses all three of these limitations. Notable prior work applied deep reinforcement learning to localize brain tumors with radiologist eye tracking points, which limits the state-action space. Here, we generalize Deep Q Learning to a gridworld-based environment so that only the images and image masks are required.

**Methods:**

We trained a Deep $Q$ network on 30 two-dimensional image slices from the BraTS brain tumor database. Each image contained one lesion. We then tested the trained Deep Q network on a separate set of 30 testing set images. For comparison, we also trained and tested a keypoint detection supervised deep learning network on the same set of training/testing images.

**Results:**

Whereas the supervised approach quickly overfit the training data and predictably performed poorly on the testing set (11% accuracy), the Deep $Q$ learning approach showed progressive improved generalizability to the testing set over training time, reaching 70% accuracy.

**Conclusion:**

We have successfully applied reinforcement learning to localize brain tumors on 2D contrast-enhanced MRI brain images. This represents a generalization of recent work to a gridworld setting naturally suitable for analyzing medical images. We have shown that reinforcement learning does not over-fit small training sets, and can generalize to a separate testing set.

## Introduction

Recently, reinforcement learning (RL, used interchangeably with the term deep reinforcement learning) has shown tremendous promise for landmark localization. Researchers have recently applied RL successfully to landmark or lesion localization in various image types and modalities [1–4]. Examples of applications are localization of breast lesions [5], lung nodules [6], anatomic landmarks on cardiac MRI [7] and vessel centerline tracing [8]. However, not much work has been done in the field of brain lesion localization with RL.

In recent work [9], Stember and Shalu applied RL to localize brain tumors on MRI. They sought to address three key shortcomings in current supervised deep learning approaches:

1. Requirement of large amounts of expert-annotated data.
2. Lack of generalizability, making it “brittle” and subject to grossly incorrect predictions when even a small amount of variation is introduced. This can occur when applying a trained network to images from a new scanner, institution, and/or patient population [10, 11].
3. Lack of insight or intuition into the algorithm, thus limiting confidence needed for clinical implementation and curtailing potential contributions from non-AI experts with advanced domain knowledge (e.g., radiologists or pathologists) [12, 13].

Their initial proof-of-principle application of RL to medical images used 2D slices of image volumes from the publicly available 2014 BraTS primary brain tumor database [14]. These T1-post-contrast images included one tumor per image. In addition, their images included an overlay of eye tracking gaze points obtained during a previously performed simulated image interpretation. The state-action space was limited to the gaze plots, consisting of the gaze points for that image. The gaze plots were essentially one-dimensional, and the various possible agent states were defined by location along the gazeplot. Actions were defined by the agent moving anterograde versus retrograde along the gaze plot, or by staying still. As a localization task, the goal was for the agent to reach the lesion. Using the manually traced tumor mask images, a reward system was introduced that incentivized finding and staying within the lesion, and discouraged staying still while the agent was still outside the tumor [9].

The results from this study showed that RL has the potential to make meaningful brain lesion localization predictions based on very small data sets (in this case, 70 training set images). Supervised deep learning woefully overfit the training set, with unsurprisingly low accuracy on the testing set (around 10%). In contrast, RL improved steadily with more training, ultimately predicting testing set image lesion location with over 80% accuracy [9].

However, the system studied was not generalized, as it included eye tracking points, which are usually not available with radiological images. Additionally, the eye tracking points confined the state-action space to one dimension. In order to apply RL more generally to medical images, we must be able to analyze raw images along with accompanying image masks without the need for eye tracking gaze plots.

In this study, we generalize the approach to show that RL can effectively localize lesions using a very small training set using the gridworld framework, which requires only raw images and the accompanying lesion masks. This represents an important early step in establishing that RL can effectively train and make predictions about medical images. This can ultimately be extended to 3D image volumes and more sophisticated implementations of RL. Gridworld is a classic, paradigmatic environment in RL [15]. Given their pixelated character, medical images tiled with a gridworld framework provide a natural, readily suitable environment for our implementation.

## Methods

### Basic terms

Following the basic approach of recent work [9], we analyzed 2D image slices from the BraTS 2014 public brain tumor database [14]. Since these are publicly available images with no patient identifying information, this study did not require IRB approval. These slices were randomly selected from among T1-weighted contrast-enhanced image slices that included brain tumor. Images from around the level of the lateral ventricles were selected. We used the BraTS 2014 data set specifically because it had used in an earlier, less generalized study using eye tracking points [9]. We wished to minimize the variables/confounders between these two studies.

As in the recent work, we implementated a combination of standard $TD(0)$ $Q$-learning with Deep $Q$ learning (DQN). The key difference was how we defined the environment, states, and actions.

**Fig. 1:**
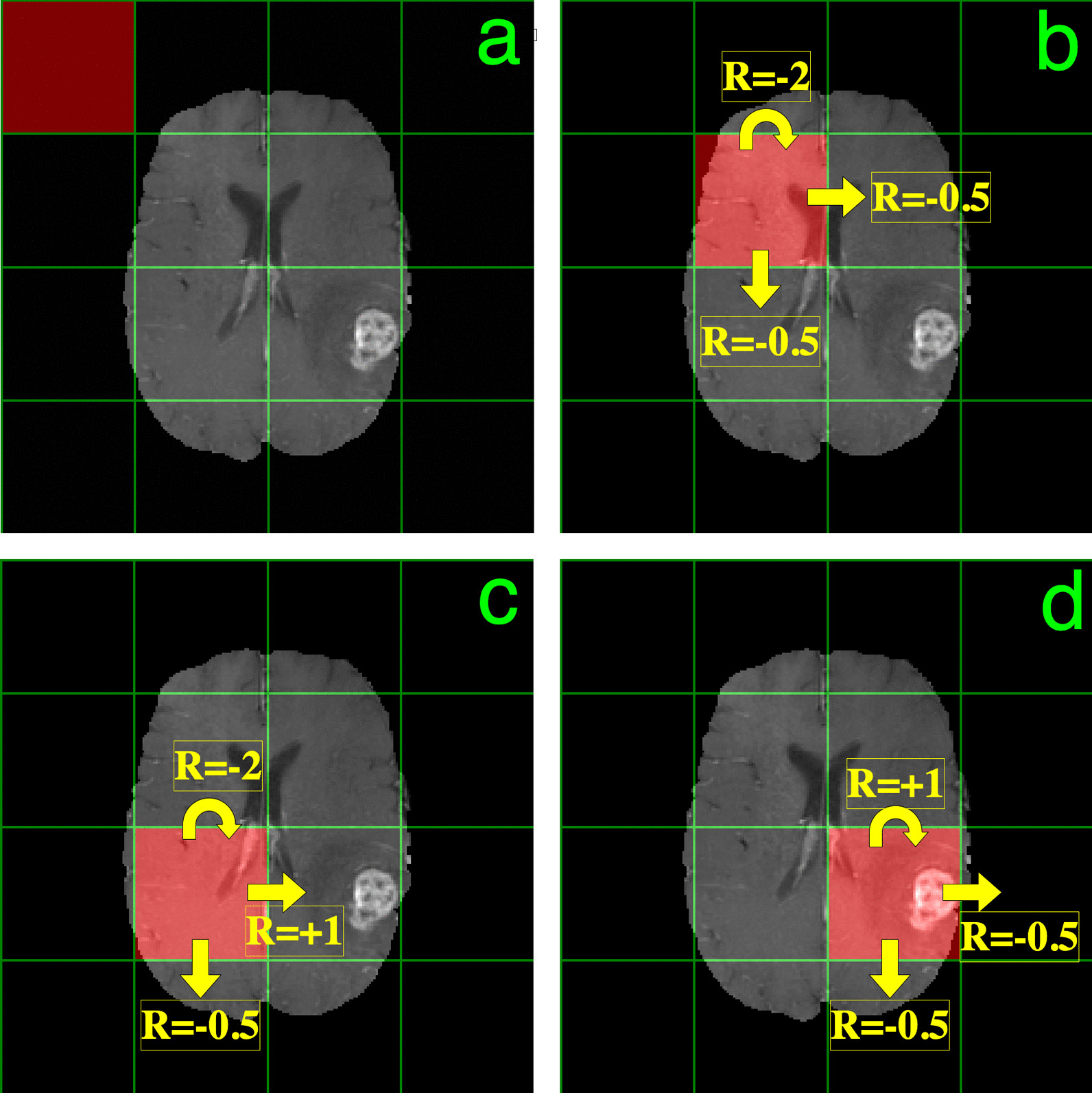
Environment and reward scheme for training. **a** Shows the initial state (${s}_{1}$) for all episodes, with the agent in the upper left corner. **b**–**d** Display the rewards in different states for the three possible actions. When the agent is not in a position overlapping or next to the lesion (b), staying in place gets the biggest penalty (reward of − 2), with a lesser penalty if the agent moves (reward of − 0.5). **c** Shows the rewards for the possible actions in the state just to the left of the mass. Moving toward the lesion so that the agent will coincide with it receives the largest possible and only positive reward (+ 1). **d** Shows the state with the agent coinciding with the lesion. Here we want the agent to stay in place, and thus reward this action with a + 1 reward

**Fig. 2:**
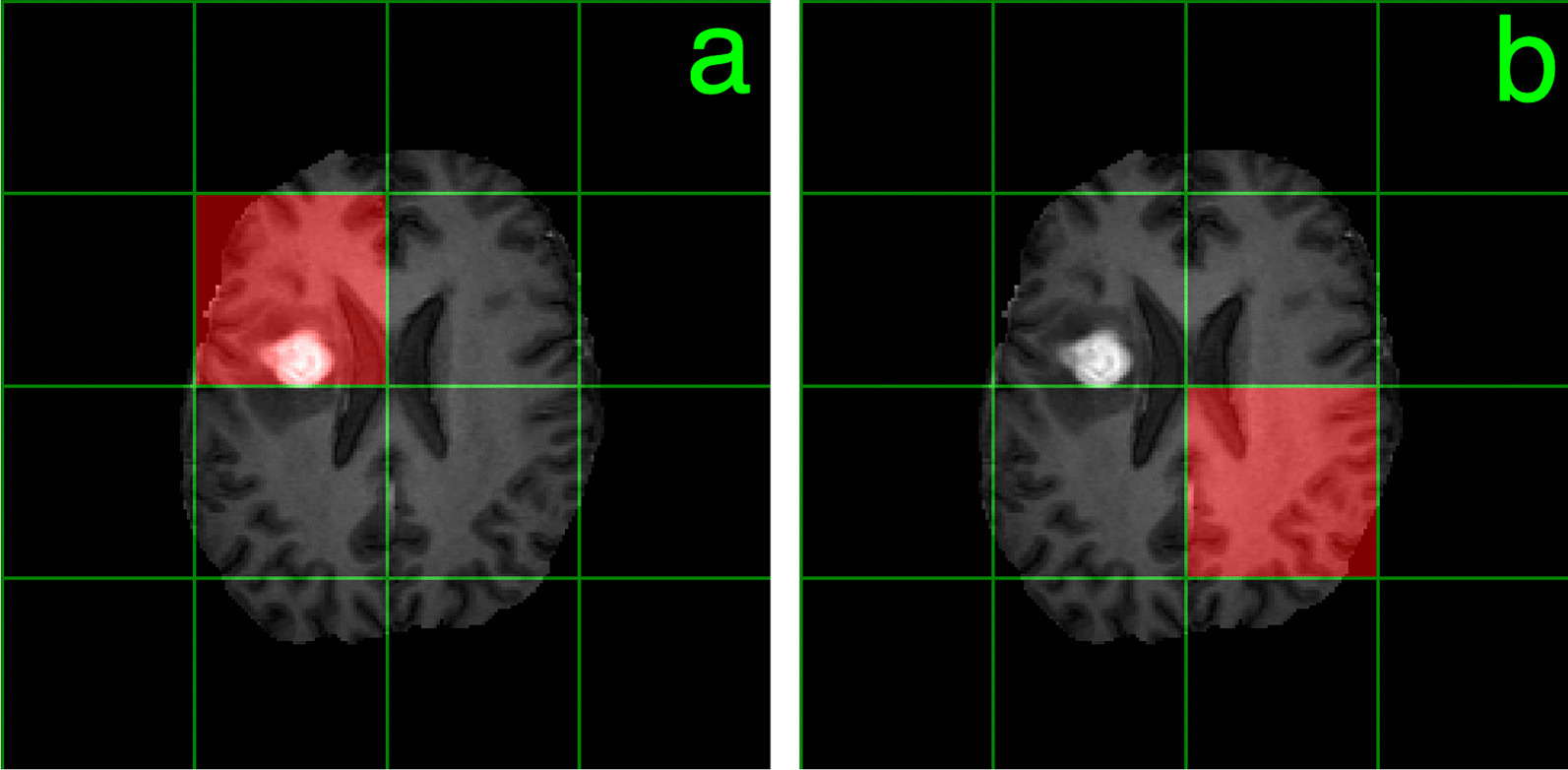
Two possible testing/deployment results. **a** shows a case of an accurate prediction, a true positive. After the 20 steps of forward inference on a presumed testing set image, the agent overlies the lesion. **b** shows a testing set miss, a false positive, where the agent does not overlap the lesion. In this particular case, there is no way for the lesion to get back to the lesion, since only the three actions of stay in place, move down and move to the right are defined in our formulation, although a more general formulation with 5 directions is possible in future work

We divided the image space of the $240\times 240$ pixel images by grids spaced 60 pixels, so that our agent occupied the position of a $60\times 60$ pixel block, shown in Figs. 1 and 2. The initial state for training and testing images was chosen to be the top-left block (Fig. 1a). The action space consisted of: (1) staying at the same position, (2) moving down by one block, or (3) moving to the right by one block. In other words, introducing some notation, the action space $\mathcal{A}\in {\mathbb{N}}_{0}^{3}$, consisting of three non-negative integers, is defined by:

1 $$\mathcal{A}=\left(\begin{array}{c}1\\ 2\\ 3\end{array}\right)=\left(\begin{array}{c}\mathrm{stay\,still}\\ \mathrm{move\,down}\\ \mathrm{move\,right}\end{array}\right).$$

To each of the possible actions, $a\in A$, given a policy $\pi$, there is a corresponding action value depending on the state, ${Q}^{\pi }(s,a)$, defined by:

2 $${Q}^{\pi }\left(s,a\right)={E}_{\pi }\left\{{R}_{t}|{s}_{t}=s,{a}_{t}=a\right\}={E}_{\pi }\left\{\sum_{k=0}^{\infty }{\gamma }^{k}{r}_{t+k+1}|{s}_{t}=s,{a}_{t}=a\right\}$$

where ${R}_{t}$ is the total cumulative reward starting at time $t$ and ${E}_{\pi }\left\{\sum_{k=0}^{\infty }{\gamma }^{k}{r}_{t+k+1}|{s}_{t}=s,{a}_{t}=a\right\}$ is the expectation for ${R}_{t}$ upon selecting action $a$ in state $s$ and subsequently picking actions according to $\pi$ [15].

### Training: sampling and replay memory buffer

In each of the ${N}_{\mathrm{episodes}}=90$ episodes of training, we sampled randomly from the $30$ training set images. For each such image we subdivided into grids, and initialized such that the first state ${s}_{1}$ was in the upper left block (Fig. 1a). We selected each action ${a}_{t}$ at time step $t$ according to the off-policy epsilon-greedy algorithm, which seeks to balance exploration of various states with exploiting the known best policy, according to

3 $${a}_{t}=\left\{\begin{array}{ll}ma{x}_{a\in A}\,\{{Q}_{t}(a)\}& \mathrm{with\,probability\, }\epsilon \\ \mathrm{random\,action\,in\, }A& \mathrm{with\,probability\, }1-\epsilon .\end{array}\right.$$

for the parameter $\epsilon <1$. We used an initial $\epsilon$ of $0.7$ to allow for adequate exploration. As $Q$ learning proceeds, and we wished to increasingly favor exploitation of a better known and more optimal policy, we set $\epsilon$ to decrease by a rate of $1\times {10}^{-4}$ per episode. The decrease continued down to a minimum value ${\epsilon }_{\mathrm{min}}=1\times {10}^{-4}$, so that some amount of exploring could always take place.

Our reward scheme is illustrated for sample states in Figs. 1b–d. The reward ${r}_{t}$ is given by

4 $${r}_{t}=\left\{\begin{array}{l}-2,\mathrm{ if\,agent\,is\,outside\,the\,lesion\,and\,staying\,still}\\ +1,\mathrm{ if\,agent\,overlaps\,the\,lesion\,and\,is\,staying\,still}\\ -0.5,\mathrm{ if\,agent\,moves\,to\,a\,position\,outside\,the\,lesion}\\ +1,\mathrm{ if\,agent\,moves\,to\,a\,position\,overlapping\,the\,lesion}.\end{array}\right.$$

### Replay memory buffer

In general, for each time $t$ we thus have state ${s}_{t}$, the action we have taken ${a}_{t}$, for which we have received a reward ${r}_{t}$ and which brings our agent to the new state ${s}_{t+1}$. We store these values in a tuple, called a transition, as ${\mathcal{T}}_{t}=({s}_{t},{a}_{t},{r}_{t},{s}_{t+1})$. For each successive time step, we can stack successive transitions as rows in a transition matrix ${\mathbb{T}}$. We do so up to a maximum size of ${N}_{\mathrm{memory}}=\mathrm{15,000}$ rows. These represent the replay memory buffer, which allows the CNN that predicts $Q$ values to sample and learn from past experience sampling from the environments of the various training images. Then, we use ${\mathbb{T}}$ to train the CNN and perform $Q$ learning [15]. The value of ${N}_{\mathrm{memory}}=\mathrm{15,000}$ was chosen to be as large as possible without overwhelming the available RAM.

### Training: Deep ${\varvec{Q}}$ network and ${\varvec{Q}}$ learning

**Fig. 3:**
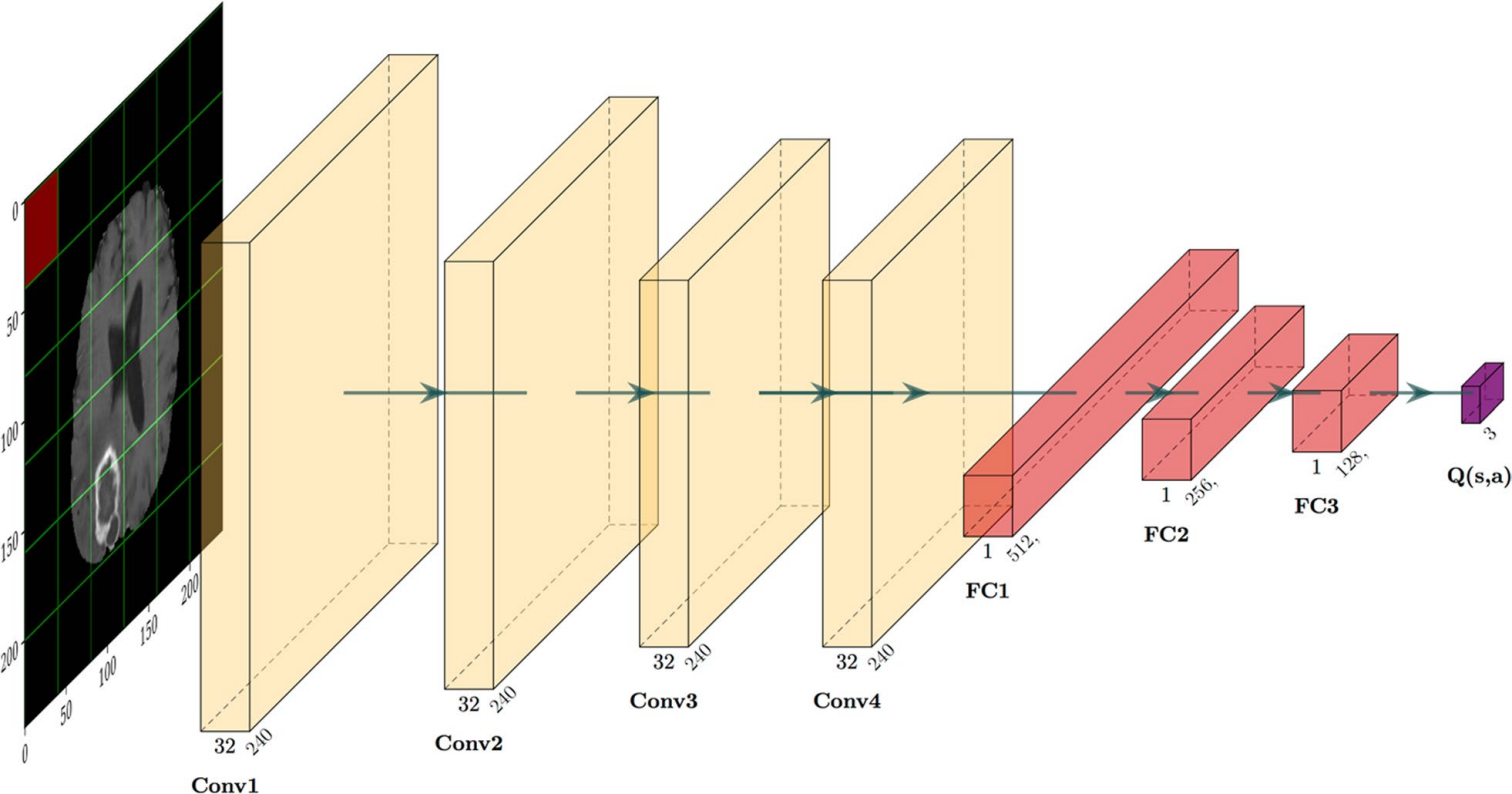
Deep Q network architecture. Of note, the output consists of the three Q values corresponding to the three possible actions. A sample input image representing the initial state ${s}_{1}$ is also noted

Using a CNN to approximate the function ${Q}_{t}(a)$, we give the CNN the name of Deep $Q$ network (DQN). The architecture of the DQN, shown in Fig. 3, is very similar to that of recent work [9]. It takes the state as input, using $3\times 3$ kernels with stride of $2$ and padding such that the resulting intermediate output layer sizes are unchanged. We produced $32$ intermediate output channels at each convolution block. The network consisted of four such convolutional layers in sequence, using exponential linear unit (elu) activation. The last output volume was flattened and followed by a $512$-node layer with elu activation, followed by a few more fully connected layers and ultimately to a $3$-node output layer representing the $3$ actions and corresponding $Q$ values.

Our DQN loss is the difference between the $Q$ values resulting from a forward pass of the DQN, which at time step $t$ we shall denote as ${Q}_{\mathrm{DQN}}$, and the “target” $Q$ value, ${Q}_{\mathrm{target}}$, computed by the Bellman equation [15]. The latter updates by sampling from the environment and experiencing rewards. Denoting the forward pass by ${F}_{\mathrm{DQN}}$, we can obtain state-action values by

5 $${Q}_{\mathrm{DQN}}^{(t)}={F}_{\mathrm{DQN}}({s}_{t}).$$

But the function approximation we wish to learn is for the *optimal* state-action values, which maximize expected total cumulative reward. We do so by $Q$ learning, in which the model learns from sampled experience, namely individual state-action pair-associated rewards. The method used in recent work [9], and that we employ here, is temporal difference in its simplest form: $TD(0)$. With $TD(0)$ the state-action value function is updated in each step of sampling to compute the $TD(0)$ target, denoted by ${Q}_{\mathrm{target}}^{(t)}$ [15]:

6 $${Q}_{\mathrm{target}}^{(t)}={r}_{t}+\gamma ma{x}_{a}Q({s}_{t+1},a),$$

where $\gamma$ is the discount factor and $ma{x}_{a}Q({s}_{t+1},a)$ is another way of writing the state value function $V({s}_{t+1})$. The key part of the environment sampled is the reward value ${r}_{t}$. Over time, with this sampling, ${Q}_{\mathrm{target}}^{(t)}$ converges toward the optimal $Q$ function, ${Q}^{\star }$. In our implementation, for each episode, the agent was allowed to sample the image for $20$ steps. We set $\gamma =0.99$, a frequently used value that, being close to $1$, emphasizes current and next states but also includes those further in the future.

### Training: Backpropagation of the Deep ${\varvec{Q}}$ network

In each step of DQN backpropagation, we randomly selected a batch size of ${N}_{\mathrm{batch}}=128$ transitions from the rows of ${\mathbb{T}}$ and computed corresponding ${Q}_{\mathrm{target}}^{(t)}$ and ${Q}_{\mathrm{DQN}}^{(t)}$ values, yielding the vectors ${\overrightarrow{Q}}_{\mathrm{target}}=\{{Q}_{\mathrm{target}}^{(t)}{\}}_{t=1}^{{N}_{\mathrm{batch}}}$ and ${\overrightarrow{Q}}_{\mathrm{DQN}}=\{{Q}_{\mathrm{DQN}}^{(t)}{\}}_{t=1}^{{N}_{\mathrm{batch}}}$. We backpropagated to minimize the loss ${L}_{\mathrm{batch}}$ of said batch,

7 $${L}_{\text{batch}}=\frac{1}{{N}_{\text{batch}}} \sum_{i=1}^{{N}_{\text{batch}}}\left|{Q}_{\text{target}}^{(i)}-{Q}_{\text{DQN}}^{(i)}\right|.$$

Fortunately, as we proceed in training ${Q}_{\mathrm{DQN}}^{(t)}$ to successively approximate ${Q}_{\mathrm{target}}^{(t)}$, our CNN function approximation should converge toward that reflecting the optimal policy, so that

8 $$\underset{t\to \infty }{\mathrm{lim}}\left({Q}_{\mathrm{DQN}}^{(t)}\right)=\underset{t\to \infty }{\mathrm{lim}}\left({Q}_{\mathrm{target}}^{(t)}\right)={Q}^{\star }.$$

To train the DQN, we employed the Adam optimizer with learning rate of $1\times {10}^{-4}$. We implemented DQN training in the Pytorch package in Python 3.7 executed in Google Colab.

## Results

We trained the DQN on $30$ two-dimensional image slices from the BraTS database at the level of the lateral ventricles. We did not employ any data augmentation. Training was performed for $90$ episodes. For each of the separate $30$ testing set images, the trained $DQN$ was applied to the initial state with agent in the top left corner and successively to each subsequent state for a total of $20$ steps. Even if the agent overlaps the lesion before the 20^th^ step, we would expect that, with adequate prior training, the agent would stay on the lesion, given the training incentive to do so, as shown in Fig. 1d. Upon testing, the agent does not have prior knowledge about where the lesion is, so we felt that taking 20 steps was adequate to reach any lesion given our 4 $\times$ 4 (16 patches) grid.

Figure 2 shows the two possible testing/deployment outcomes. Figure 2a displays a true positive (TP) outcome, in which the agent overlies a patch that has nonzero overlap with the lesion. Figure 2b shows a false positive (FP) missed testing set case, in which the agent has zero overlap with the lesion after 20 steps of deployment.

Because this is a localization task, we take both true and false negative to be zero. Hence our accuracy is defined as $\frac{TP}{TP+FP}$.

In order to compare the performance of RL / Deep $Q$ learning with that of standard supervised deep learning, we trained a localization supervised deep learning network as well. More specifically, we trained a keypoint detection CNN with architecture essentially identical to that of our DQN. Again, to make the comparison as fair as possible, we trained the keypoint detection CNN on the same $30$ training images for $90$ epochs. TP is defined when the keypoint lies within the lesion, FP when it lies outside of the lesion. Not surprisingly for such a small training set, the supervised keypoint detection CNN quickly overfit the training set, with training and testing set losses diverging before the $10$ th epoch.

Figure shows a head-to-head comparison of the two techniques. It plots accuracy of the trained networks on the separate testing sets of 30 images as a function of training time, measured as episodes for deep reinforcement learning and as epochs for supervised deep learning. Supervised deep learning does not learn in a way that generalizes to the testing set, as evidenced by the essentially zero slope of the best fit line during training. The DQN learns in a more generalized manner during training, as manifested by the positive slope of the best fit line. Ultimately, RL/Deep $Q$ learning achieves an average accuracy of $70$% over the last $20$ episodes, whereas supervised deep learning has a corresponding mean accuracy of $11$%, a difference that is statistically significant by standard $t$-test, with $p$-value of $5.9\times {10}^{-43}$.

We also note that if the training:testing split were more weighted toward training images, for example 55:5, we would expect the following: a testing set accuracy of 3/5 or 4/5 could very well be due to chance. In this case, for better statistics, one would perform 12-fold cross validation and take the average testing set accuracy, computing a confidence interval.

In our case, the training:testing split was 30:30. As such, it stands to reason that 21/30 = 0.7 or 70% testing set accuracy is very unlikely due to chance. Nevertheless, we ran twofold cross validation, reversing training and testing set, and obtained a testing set accuracy again of 70%.

## Discussion

We have successfully applied deep reinforcement learning, here implemented as Deep $Q$ learning, in tandem with temporal difference learning. Specifically, in this demonstration, we have applied the approach to identifying and locating glioblastoma multiforme brain lesions on contrast-enhanced MRI. By locating, we mean more specifically locating at least one point within the lesion, noting that the lesion can have nonzero overlap with more than one patch in our gridworld image tiling.

We have shown that the approach can produce reasonably accurate results with a training set size of merely $30$ images. This number is at least an order of magnitude below what is generally considered necessary for radiology AI. This follows from the fact that current radiology AI has been dominated by supervised deep learning, an approach that depends on large amounts of annotated data. Supervised deep learning typically requiring hundreds (or, preferably, thousands) of annotated images to achieve high performance.

To restate the three key limitations of the currently prevalent supervised deep learning approach in radiology, they are: (1) Requirement of large amounts of expert-annotated data; (2) Susceptibility to grossly incorrect predictions when applied to new data sets; and (3) Lack of insight or intuition into the algorithm.

This proof-of-principle work provides evidence that reinforcement learning can address limitation #1. It can also address limitation #3, as evidenced by the reward structure illustrated in Fig. 1b–d.

We note that deep reinforcement learning becomes very time-consuming to train for large training sets, making this less practical for our proof-of-principle work. We note further that the purpose of this work was to show the data efficiency of deep reinforcement learning, which would not be furthered by training it on a large database. Prior work has shown [16] that for 700 training set images, supervised deep learning can achieve high accuracy, of around 92%. Hence, the transition to accurate supervised deep learning regression on the BraTS database can be presumed to lie somewhere between 30 and 700 training set images.

Future work will address limitation #2 by comparing deep reinforcement learning and supervised deep learning trained on data from one institution and tested on separate images from another institution, ideally constituting a wide range of tumor characteristics.

**Fig. 4:**
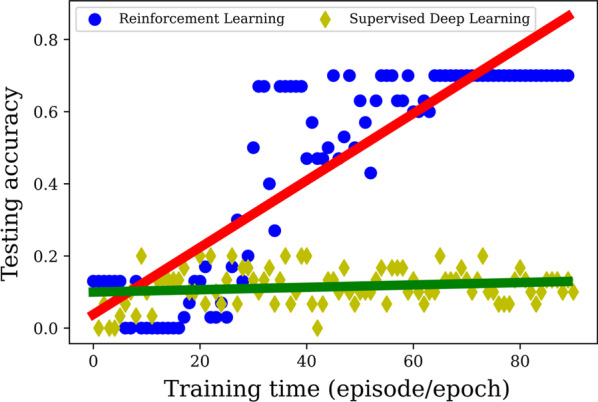
Comparison between (deep) reinforcement learning/Deep Q learning and supervised deep learning. The mean accuracy of the testing set of 30 images is plotted as a function of training time, which is measured for reinforcement learning in episodes, and for supervised deep learning in epochs. Both methods were trained on the same 30 training set images. Blue circles represent the results of reinforcement/Deep Q learning, with best fit line in red. Accuracy for supervised deep learning is shown as yellow diamonds, with best fit line in green. Whereas supervised deep learning quickly overfits the small training set and thus cannot learn from it in any generalized manner, the Deep Q network is able to learn over time in a way that can generalize to the separate testing set

An important limitation of the present work is that it has been performed on two-dimensional image slices. Future work will extend to fully three-dimensional image volumes. As can be seen in Fig. 4, the Deep $Q$ learning training process is somewhat noisy. Future work will utilize different techniques in reinforcement learning to learn in a smoother fashion. It should be noted that we tried employing policy-gradient learning to achieve this less noisy learning. We did so with the actor-critic approach in both its single-agent version, A2C, and its multi-agent form, A3C. Both approaches failed to learn as Deep $Q$ learning could. We suspect that this is caused by the sequential nature of sampling in A2C/A3C, which could not make use of the varied sampling across environments (i.e., different training set images) and states. We anticipate that incorporating a replay memory buffer with policy gradient may ultimately work best, and this will be a focus of future work.

## Conlusions

We have shown as proof-of-principle that deep reinforcement learning can accurately localize brain lesions on MRI using a gridworld framework. High testing set accuracy is achieved despite a very small training set. Hence, deep reinforcement learning can provide a data-efficient method to localize lesions when limited image data is available.

## Data Availability

The BraTS database can be accessed at: http://braintumorsegmentation.org/

## Abbreviations

RL: Reinforcement learning
DRL: Deep reinforcement learning (used interchangeably with reinforcement learning)
DQN: Deep Q Network
SDL: Supervised deep learning
